# Transcriptome sequencing of the Microarray Quality Control (MAQC) RNA reference samples using next generation sequencing

**DOI:** 10.1186/1471-2164-10-264

**Published:** 2009-06-12

**Authors:** Shrinivasrao P Mane, Clive Evans, Kristal L Cooper, Oswald R Crasta, Otto Folkerts, Stephen K Hutchison, Timothy T Harkins, Danielle Thierry-Mieg, Jean Thierry-Mieg, Roderick V Jensen

**Affiliations:** 1 Virginia Bioinformatics Institute, Virginia Tech, Blacksburg, VA 24061, USA; 2 454 Life Sciences, Inc., 20 Commercial Street, Branford, CT 06405, USA; 3 Roche Applied Science, Indianapolis, IN 46250, USA; 4 National Center for Biotechnology Information, National Library of Medicine, National Institutes of Health, 8600 Rockville Pike, Bethesda, MD 20894, USA; 5 Department of Biological Sciences, Virginia Tech, Blacksburg, VA 24061, USA

## Abstract

**Background:**

Transcriptome sequencing using next-generation sequencing platforms will soon be competing with DNA microarray technologies for global gene expression analysis. As a preliminary evaluation of these promising technologies, we performed deep sequencing of cDNA synthesized from the Microarray Quality Control (MAQC) reference RNA samples using Roche's 454 Genome Sequencer FLX.

**Results:**

We generated more that 3.6 million sequence reads of average length 250 bp for the MAQC A and B samples and introduced a data analysis pipeline for translating cDNA read counts into gene expression levels. Using BLAST, 90% of the reads mapped to the human genome and 64% of the reads mapped to the RefSeq database of well annotated genes with e-values ≤ 10^-20^. We measured gene expression levels in the A and B samples by counting the numbers of reads that mapped to individual RefSeq genes in multiple sequencing runs to evaluate the MAQC quality metrics for reproducibility, sensitivity, specificity, and accuracy and compared the results with DNA microarrays and Quantitative RT-PCR (QRTPCR) from the MAQC studies. In addition, 88% of the reads were successfully aligned directly to the human genome using the AceView alignment programs with an average 90% sequence similarity to identify 137,899 unique exon junctions, including 22,193 new exon junctions not yet contained in the RefSeq database.

**Conclusion:**

Using the MAQC metrics for evaluating the performance of gene expression platforms, the ExpressSeq results for gene expression levels showed excellent reproducibility, sensitivity, and specificity that improved systematically with increasing shotgun sequencing depth, and quantitative accuracy that was comparable to DNA microarrays and QRTPCR. In addition, a careful mapping of the reads to the genome using the AceView alignment programs shed new light on the complexity of the human transcriptome including the discovery of thousands of new splice variants.

## Background

The goal of the Microarray Quality Control (MAQC) project was to identify quality metrics for evaluating gene expression measurement technologies[1]. The MAQC study provided a set of reference RNA samples with large numbers of differentially expressed genes consisting of the commercially available A sample from pooled human cell lines and the B sample from a pooled human brain preparation. These two samples were exhaustively analyzed on a number of different whole genome microarray platforms and Quantitative Real-Time PCR (QRTPCR)[1,2].

With the advent of the new "next-generation" sequencing technologies, it is now practical to analyze gene expression by the direct shotgun sequencing of complementary DNA (cDNA) synthesized from RNA samples[3,4]. This approach has already been applied to discover novel gene sequences in the transcriptomes of human cell lines[5] and plants[6,7] and to identify cancer related Single Nucleotide Polymorphisms (SNPs) in transcribed sequences in human cancer transcriptomes[8]. More recently papers have appeared using this technique to confirm predicted genes in the bacteria[9] and to identify novel transcription products and to quantify expression levels in the yeast[10,11], mouse [12-14], human Hela cells[15], and the cerebellar cortex of schizophrenic patients[16].

To provide a preliminary evaluation of the performance of these promising technologies for gene expression analysis, we performed deep sequencing of the Microarray Quality Control (MAQC) reference RNA samples using Roche's 454 Genome Sequencer FLX (GS FLX)[17]. For this study, we generated more than 3.6 million sequence reads of average length 250 bp for cDNA generated from the MAQC A and B samples[1]. Using our ExpressSeq pipeline over 90% of the reads mapped to the human genome and 64% of the reads mapped to the RefSeq database of well annotated genes using BLAST with e-values ≤ 10^-20^, corresponding to at least 50 perfect match bases. (Because of the long read lengths, more stringent e-values, eg. 10^-50 ^or 10^-100^, could also be used for the BLAST searches, however these would begin to miss reads that partially hit exons that are not already contained in RefSeq.) By counting the numbers of reads that map to individual genes we can measure the gene expression levels in the two samples to evaluate the MAQC quality metrics and compare the results with DNA microarrays and QRTPCR.

We have also performed a careful mapping of the transcriptome reads to the genome using the AceView alignment programs used to construct the AceView database of genes and alternative splice variants from cDNA sequences in all public databases[18]. Many of the GS FLX reads spanned one or more exon junctions recovering approximately 60% of the exon junctions already annotated in the RefSeq database and 36% of the exon junctions in the comprehensive AceView database. In addition thousands of new exon junctions were identified spanning standard introns in the genome that have not been previously annotated in any public databases. These additional results speak to the great promise of deep transcriptome sequencing to rapidly shed new light on the complexity of the eukaryotic transcriptome.

## Results

### Mapping of reads to the RefSeq, CCDS, and AceView Databases

Complementary DNA (cDNA) for the MAQC A and B reference RNA samples was shotgun sequenced in 11 separate sequencing runs on the GS FLX platform. A total of 3.6 million transcriptome reads were generated of average length 250 bp, 1.7 million for Sample A and 1.9 million for Sample B. Two different protocols were compared in this study for the preparation of cDNA from the RNA samples: 1) ODT: an OligoDT reverse transcription (RT) protocol to generate double stranded cDNA which was then converted into a single-stranded library for sequencing and 2) TSEQ: a random primer RT process directly to single-stranded cDNA library for Transcriptome Sequencing. [See Additional file 1 for Supplementary Methods for full details.]

The reads were mapped to the human genome and to the RefSeq database of well-annotated RNA sequences using the ExpressSeq pipeline implemented on a Windows Desktop. To evaluate the coverage of the expressed sequences, only the best BLAST alignment with the smallest e ≤ 10^-20 ^value was counted as a hit. Table 1 provides a summary of these mapping results for both the ODT and TSEQ protocols. Overall more than 90% of the reads mapped to genomic DNA, while only 58–73% of the reads mapped to RefSeq gene sequences, indicating that there are still many more expressed sequences to be included in RefSeq. Only a small number of reads (3–10%) failed to map to the human genome with e ≤ 10^-20^. Although some of these unmappable reads may be poor quality reads or sequencing artifacts, many may still represent novel spliced exons or edited transcripts worthy of further study.

**Table 1:** ExpressSeq read counts that hit* NCBI databases

	**TSEQ**	**TSEQ**	**ODT**	**ODT**
	**MAQC A**	**MAQC B**	**MAQC A**	**MAQC B**
Total # of Reads	823,575	1,079,508	881,555	846,304
Reads that Hit Human Genomic DNA	89%	96%	97%	96%
Reads that Hit Human RefSeq (38,698 sequences)	60%	58%	73%	67%
Reads that Hit Human NM's (24,654 sequences)	53%	50%	59%	45%
Reads that Hit CCDS (17,751 sequences)	31%	23%	35%	20%
Reads that Hit AceView (278,760 sequences)	77%	85%	83%	84%
Orphan Reads (Hit none of the above)	10%	4%	3%	4%

*An ExpressSeq "hit" is the single best BLAST alignment of the GS FLX reads to sequences in each database with e ≤ 10^-20^.

Of the 2.3 million reads that mapped to the RefSeq sequences, approximately 80% aligned to the well annotated mRNA sequences denoted with an NM identifier while the remaining RefSeq reads hit the predicted XM mRNA sequences or the well annotated or predicted regulatory sequences designated by NR and XR, including ribosomal rRNA. In addition, approximately 60% of the pooled cell line NM reads and 45% of the brain NM reads mapped to the protein coding regions identified in the Consensus Coding Sequences (CCDS) database which suggests that transcripts in the brain tend to have longer 3' UTRs.

Finally, since the AceView databases of expressed transcripts provides the most comprehensive listing of spliced and unspliced human mRNAs compiled from all public cDNA sequences, we applied the ExpressSeq pipeline to count all reads that hit sequences in the AceView database of 278,260 "good" genes and their splice variants that are supported by at least 6 overlapping GenBank sequences or encode a putative protein. Table 1 shows that an additional 10 – 27% of the total reads hit at least one of these AceView genes and splice variants that are not yet described by the RefSeq.

### Depth and breadth of gene coverage

**Figure 1:**
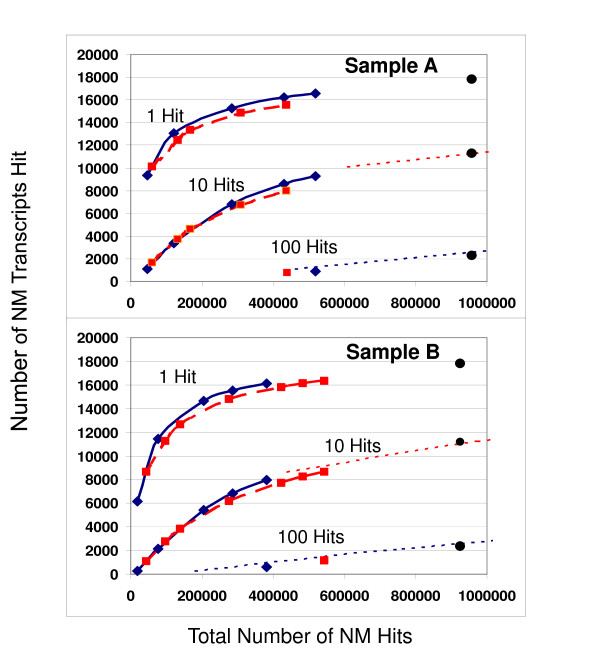
**Depth of transcriptome sequencing coverage**. The number of RefSeq NM genes detected by transcriptome sequencing increases with the total number of reads that hit any NM gene, *N*, accumulated in multiple sequencing runs. The coverage curves, *C*, show how the number of genes detected approaches saturation for (**a**) the A sample and (**b**) the B sample as the total number of reads that align to any NM gene (with e ≤ 10^-20^) increases using both the ODT (blue diamonds, solid line) and TSEQ (red squares, dashed line) methods of sample preparation. The figures also show the numbers of genes that receive at least 10 and 100 BLAST hits from the GS FLX reads (for ~1× sequence coverage of a typical 2500 bp mRNA). The single points at the far right of the figures show the combined results from both sample preparation methods. Since the coverage function for random sampling obeys the approximate scaling relationship, *C*_*n*_*(N) ~C*_*n*/*x*_*(N/x)*, where *n *is the minimum number of hits, the coverage curve for 10 hits can be predicted from the empirical results for 1 hit, and the coverage curve for 100 hits from the results for 10 hits, as indicted by the dotted curves. [See Additional file 1**for Supplementary Analysis**]

Since the shotgun sequencing procedure is expected to provide a random sampling of the different cDNA strands, most of the reads will hit the most abundant genes. Consequently, more sequencing reads must be generated to detect the less abundant genes. Fig. 1a and Fig. 1b show how increasing numbers of genes are detected as the number of reads is increased using multiple sequencing runs on the MAQC A and B samples. In the upper curves we have plotted the numbers of NM genes in the RefSeq database that are hit by at least 1 read as a function of the total number of reads that hit any NM genes. As the numbers of reads increase, the numbers of different NM genes detected increases rapidly and then gradually approaches saturation. [See Additional file 2 for the hit counts to the NM genes for all sequencing runs.]

These detection curves indicate that after 200,000 – 300,000 reads are generated which map well to any NM gene in the RefSeq database (corresponding to 1 full sequencing plate on the GS FLX), the detection of new genes in the A and B samples by at least 1 hit reaches a level of approximately 60% of all of the 24,654 NM genes in RefSeq. With increasing numbers of reads the detection of new genes continues to increase at a decreasing rate. Combining all of the approximately 1,000,000 mapped reads from both the ODT and TSEQ sample preparations, the depth of gene coverage increases to 73% of all NM genes for both the pooled human cell lines (A sample) and the human brain (B sample). Even with one million mapped reads the detection curves in Fig. 1 do not appear to have reached saturation. It is likely that deeper transcriptome sequencing would detect even more of the less abundant genes in these transcriptionally complex reference RNA samples.

Data points for the detection of genes with at least 10 and 100 hits are also displayed in Fig. 1 corresponding to 1× and 10× coverage of a typical 2500 bp gene. The dotted curves also indicate how the 1 hit results can be used to predict the 10 hit coverage curve and the 10 hit results predict the 100 hit curve, consistent with the assumptions of random sampling of the cDNA samples [See Additional file 1 for Supplementary Analysis] Although there are far fewer genes with 100 hits (~2000 for both the A and B samples at 1,000,000 mapped reads), there may be sufficient numbers of reads for the full assembly of gene sequences and the identification of homozygous and heterozygous SNPs for these most abundant transcripts[8].

**Figure 2:**
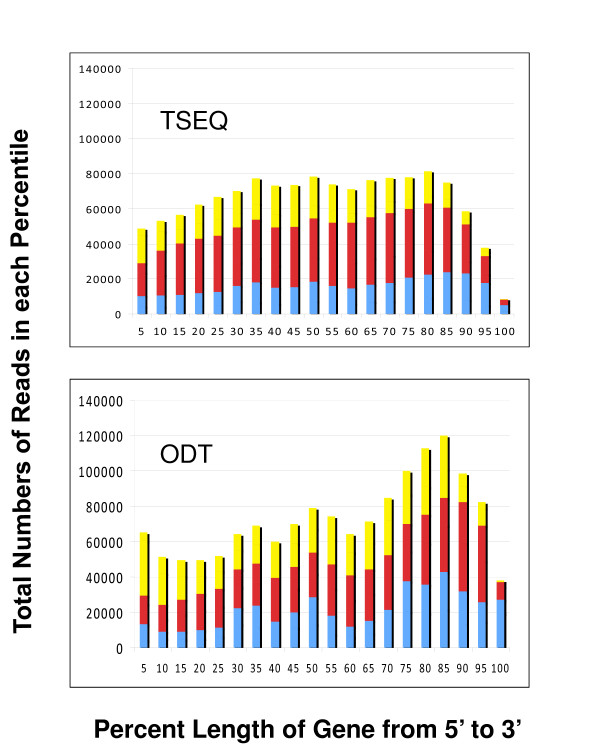
**Shotgun transcriptome sequencing covers gene sequences from the 5' to 3' ends**. The transcript coverage for both the (**a**) random primer TSEQ and (**b**) Oligo DT sample preparation protocols are compared for short (< 2,500 bp), medium (>2,500 bp and < 10,000 bp), and long (> 10,000 bp) RefSeq genes. The x axis divides the transcripts into 20 bins from the 5' to 3' end and the vertical bars plot the number of unique reads that BLAST with e ≤ 10^-20 ^into each bin for the long (blue), medium (red), and short (yellow) genes. The coverage is determined by counting the numbers of reads that align to the RefSeq sequence with 5' end in each bin. The apparent decrease of coverage at the 3' end for each method is due to reduced probability that the average 250 bp reads will start in the 3' extreme > 90% bins, for shorter genes < 2500 bp.

Fig. 1a and Fig. 1b also show a comparison of the depth of coverage provided by the two different sample preparation protocols. The ODT method appears to be slightly more sensitive in detecting rare genes than the TSEQ method, perhaps due the shorter effective gene length caused by 3' bias in the cDNA synthesis. These differences are illustrated in Fig. 2a and Fig. 2b which show the breadth of the transcript coverage for the two protocols for the long (greater than 10,000 bp), medium (between 2,500 and 10,000 bp), and short (less than 2,500 bp) transcripts. As expected the TSEQ method provides more uniform coverage of the transcripts from the 5' to 3' end for transcripts of all sizes; however, the ODT method still gives surprisingly good results even for the longest transcripts where the coverage density decreases only 50% from the 3' to 5' end.

### Reproducibility of expression levels

The number of reads that map to specific transcripts provides a direct quantitative measure of the gene expression level in the sample. Unfortunately, uncertainties in the length and uniformity of coverage of individual genes and the relative efficiencies of the reverse transcription (RT) reactions used to transform RNA into cDNA currently prevent this digital readout from being used as an absolute measure of gene expression. Nevertheless, it can still be effectively used to quantify the relative expression of transcripts in different samples. In particular, for computing gene expression ratios the dependence of hit counts on the length of the gene and the RT efficiency should factor out.

**Figure 3:**
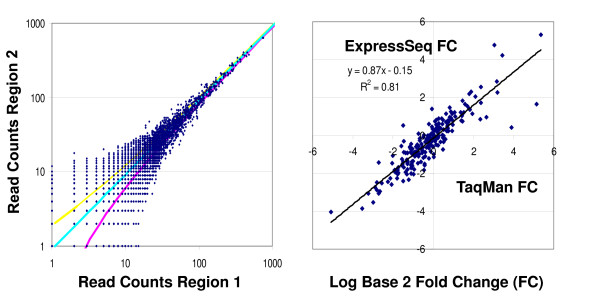
**Performance of the GS FLX transcriptome sequencing evaluated using the MAQC metrics for reproducibility and accuracy**. (**a**) The reproducibility of the ExpressSeq gene counts is displayed using a scatterplot of the numbers of unique hits for different RefSeq genes counted in two sequencing regions on the same sequencing plate using the TSEQ sample preparation method. The total numbers BLAST hits to RefSeq genes in each region were 153,697 and 139,962. The solid blue line indicates the ideal result of equal fractions of gene hits for each sequencing region and the two solid, yellow and red, curves show the median CV's ~ predicted by the Poisson sampling error model. (**b**) The relative accuracy of the ExpressSeq gene counts is illustrated with a scatterplot of the log base 2 fold-changes (FC) in gene expression levels for the MAQC A and B samples on the y-axis compared with the TaqMan measurements from the original MAQC study on the x-axis for 217 RefSeq genes that were called "present" in both samples by TaqMan and the microarray platforms. The trendline shows the best fit with Pearson correlation *R*^2 ^= 0.81.

The first metric examined in the MAQC project was reproducibility of gene expression levels[1]. For shotgun transcriptome sequencing, this metric can be evaluated by comparing the fraction of reads that hit each transcript in multiple sequencing runs and measuring the coefficient of variation (CV) of the read counts. Fig. 3a shows a typical scatter plot of the hit counts for each of the 36,698 RefSeq genes measured for the same sample preparation on two regions of the same sequencing plate. In this example, both sequencing regions had approximately the same numbers of total reads and the straight diagonal line shows the expected read counts if both sequencing regions measured equal expression levels. The results are similar when the same sample preparation is run on two different sequencing plates. However, gene specific differences do arise when comparing the counts from the two different (ODT and TSEQ) sample preparation methods which suggests that different methods of generating cDNA from RNA may provide the largest source of variability in the absolute read counts. (Data not shown.)

For the 140,000 – 150,000 reads used to generate Fig. 3a, the dynamic range spanned only 3 orders of magnitude up to ~1000 hits for the most abundantly expressed gene. However, for random sampling this dynamic range is expected to increase linearly with increasing depth of coverage, *N*. In addition, the variability from the ideal is well described by a simple Poisson sampling model for the shotgun sequencing process which predicts that the hit count CV should decrease with increasing hit number, *N*, as . Consequently, the median CV of the expression levels is expected to be < 10% for genes with *N *> 100 Since the best microarray platforms in the MAQC study achieved median CV's < 10%, this Poisson error model predicts that the level of reproducibility of the gene expression measurements from transcriptome sequencing will be comparable to the DNA microarrays for genes that have been detected by at least 100 reads. This random sampling error model, requiring no background correction, is much simpler than most error models for microarray-based gene expression data. Moreover, both the dynamic range and CV of the transcriptome gene expression measurements exhibit systematic improvement with increasing depth of coverage, *N*.

### Accuracy, sensitivity, and specificity

Additional metrics considered by the MAQC studies for the evaluation of gene expression platform performance include the three interrelated measures of accuracy, sensitivity, and specificity[1]. The relative accuracy of the gene expression levels inferred from transcriptome read counts can be assessed by comparing the fold changes of differentially expressed genes between the A and B samples with the DNA microarray and with QRTPCR results from the MAQC study. Fig. 3b shows a comparison of the fold-changes measured using transcriptome sequencing of the A and B samples with the TaqMan results for 217 RefSeq genes that were detected at sufficient levels to be called "present" in both the A and B samples by TaqMan and by all the major commercial microarray platforms (ABI, Affymetrix, Agilent, GE, and Illumina)[1,2]. This scatterplot shows that the ExpressSeq results for gene expression changes show excellent correlation with the corresponding TaqMan results with a Pearson correlation of *R*^2 ^= 0.81. By comparison the Pearson correlations for the microarray measurements with TaqMan for these same 217 genes were Affymetrix (0.75), Agilent (0.80), and Illumina (0.73). [See Additional file 1 for Supplementary Figure]

The success of sequencing based expression profiling is a result of the high sensitivity of transcriptome reads in detecting and counting these abundant genes. For each of the 217 genes called "present" in both the A and B samples on every microarray platform and individual TaqMan assays, the 3.6 million reads generated by the transcriptome sequencing gave at least one hit in both the A and B samples with a maximum of 1944 hits and a median of 55 for the A sample and a maximum of 1226 hits and a median of 46 for the B sample. These numbers translate to median read counts of ~25 reads per gene per million mapped reads. Since the detection limit for a "present" call on DNA microarrays is frequently estimated to be ~1 copy per cell[19], the ExpressSeq results are in good agreement with the estimates presented in Ref. [13] that a gene present at the level of 1 copy/cell should yield at least 3 shotgun reads per kbp per million mapped reads (RPKM) or 6 reads per (2 kbp) gene per million mapped reads.

High specificity is also necessary for accurate measures of gene expression changes since signals associated with more than one gene can mask the true expression level of a rare transcript. For example multiple reads from repeat regions such as Long Interspersed Nuclear Elements (LINEs), Short Interspersed Nuclear Elements (SINEs), and ALUs in the untranslated regions of the transcriptome can distort the true expression level. The ExpressSeq pipeline avoids this problem with a stringent requirement that each read to be counted only once for the best BLAST hit to each RefSeq. As an illustration, for a highly expressed, brain specific genes, such as the Myelin Basic Protein (MBP), there were a total of 5653 unique reads that hit the 5 annotated splice variants in RefSeq for the MAQC brain sample B while only 52 reads hit the MAQC pooled human cell lines sample A. This dynamic range of 109-fold difference compares well with the DNA microarray measurements for the same gene in the A and B samples, Affymetrix (85-fold), Agilent (210-fold), and Illumina (99-fold).

### Discovery of New Splice Variants

The AceView database of transcribed sequences was generated by co-aligning all publically available cDNA sequences generated in the past 15 years of Sanger sequencing to the human genome[18] The same alignment software was used here to successfully map 88% of the long GS FLX reads for the MAQC A and B samples to the NCBI Build 36.3 of the human reference genome with the requirement that over 90% (on average > 245 bp) of the entire sequence align perfectly.

Table 2 shows that this alignment of the MAQC reads proved very useful for the discovery of alternative spliced exons in the A and B samples. Using the AceView software we have identified 137,899 unique exon junctions. [See Additional file 3.] These were defined by the stringent requirement that two exons are separated by a standard intron with GT-AG (99.35%), GC-AG (0.6%), or AT-AC (0.05%) on either end with at least 8 consecutive bases that perfectly match the exon sequences on each side of the junction.

**Table 2:** Exon junctions* identified by alignment of the GS FLX reads from the MAQC A and B Samples to the Human Genome compared with RefSeq and AceView

	**RefSeq**	**MAQC**	**AceView**
Total Number of Exon Junctions	190,325	137,899	360,517
Number of Exon Junctions not in RefSeq	0	22,193	170,557
Number of Novel Exon Junctions not in RefSeq or AceView	0	8,432	0

*Exon junctions were identified by the requirements that the reads align to the genome separated by standard GT-AG, GC-AG, or AT-AC introns.

Using these criteria, the 3.6 million MAQC reads recovered over 60% of the 199,469 exon junctions currently supported by cDNA sequences in the March 24, 2008 release of RefSeq. In addition these GS FLX reads identified 22,193 exon junctions not yet in RefSeq. [See Additional file 4.] These new exon junctions were supported by slightly more reads from the TSEQ random primer sample preparation method (55%) and from the pooled cell line sample A (57%). Although 62% of these novel exons junctions have already been described in the comprehensive AceView database culled from all publicly available cDNA sequences (GenBank, RefSeq, dbEST, and TRACE), 8,432 appear to be novel to both RefSeq and AceView.

From the 22,193 new exon junctions we also identified 912 cassette exons defined by the requirements that the GS FLX reads support both bordering GT-AG intron-exon boundaries inside of an intron already defined by RefSeq. Although 662 were already represented in the AceView database, the GS FLX reads provided support for 250 novel cassette exons. [See Additional file 4.] In addition, the GS FLX reads identified 504 new exon junctions that skip exons in RefSeq genes and 192 of these junctions events were new to the AceView database as well. [See Additional file 4.] In this analysis, the new cassette exon and skipping exon events were only counted as alternative splicing events if other GS FLX reads also supported the conventional RefSeq junctions for these genes.

Not surprisingly, the conventional RefSeq exon junctions were biased to the more commonly expressed junctions with an overall average support of 39.1 GS FLX reads, while the additional junctions also found in AceView were supported by an average of 3.5 FLX reads and the novel candidate junctions by an average of 1.3 reads. Since the MAQC A sample consists of pooled RNA from 10 different cell lines, it might be expected that some of the novel exon junctions may arise from abnormal chromosomal arrangements in these immortalized cells. However, the B sample from normal human brain tissue contributed comparable numbers of novel exon junctions: 51% in B, 52% in A and 3% in both.

**Figure 4:**
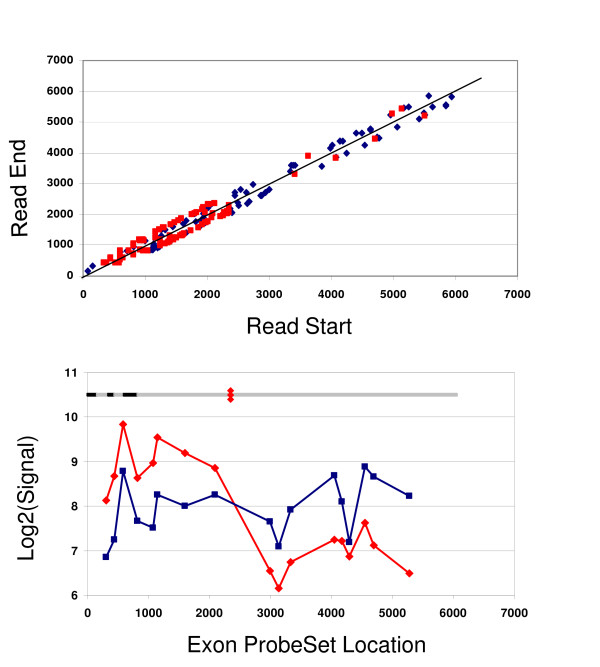
**Transcriptome sequencing detects differentially expressed transcript variants**. Because of the diverse nature of the MAQC A and B samples it is expected that there may be many genes with differentially expressed transcript variants. For example the original MAQC study found that the ELAVL1 gene was more highly expressed in Sample B than A on microarrays with probes designed to the 3' end of the gene, but more highly expressed in sample A on microarrays and the TaqMan, StaRT-PCR, and Quantigene with probes in the 5' end. Using the transcriptome sequences from both the ODT and TSEQ preparations, the differential expression of the two transcript variants in the A and B samples resulting from alternative polyadenylation sites is immediately clear. (**a**) The BLAST start and end positions of each read are plotted on the x and y for every read that hit the ELAVL1 gene in Samples A (red points) and B (blue points). Plotted this way, the forward reads correspond to the points above the gray 45 degree line and the reverse reads below. The reads for the brain Sample B cover the entire 6075 bp length of the RefSeq gene from the 5' to 3' end while most the reads for the pooled cell line Sample A hit a much shorter transcript with a truncated 3' UTR. This conclusion is also confirmed by the Affymetrix Exon 1.0 array. (**b**) The hybridization results for the core exon probesets for the ELAVL1 gene are plotted for Samples A (red points) and B (blue points) showing the differential expression of the short and long transcript variants. The black and grey bars at the top of **(b) **show the coordinates of the 6 exons of ELAVL1. The red diamonds indicate an alternative polyadenylation site in the middle of the 6^th ^exon identified by AceView.

Finally, we note that the ExpressSeq results can be used to directly measure the differential expression of both the novel and well documented transcript variants, providing a complementary platform to the new exon microarrays. For example, Fig. 4 compares the transcriptome sequencing results with the Affymetrix exon array measurements for two transcript variants of the ELAVL1 found in the original MAQC studies to be differentially expressed in the MAQC A and B samples[2].

## Discussion

Transcriptome sequencing using the massively parallel, next-generation sequencing technologies provides a high-throughput method for rapidly generating millions of reads for expressed sequences from different RNA samples[3,4,6-13,15,20]. These reads can be used to verify predicted genes and discover new transcription products and the expression levels can be quantified by direct counting of the numbers of reads mapping to different genes and transcript variants. In addition Single Nucleotide Polymorphisms (SNPs) in both the coding and noncoding regions of expressed genes can be identified by comparing the sequences of multiple matching reads with reference sequences [8]. In the past year these new technologies have already provided radically new insights into the transcriptional complexity of a number of different organisms from bacteria[9], to yeast[10,11], plants[6,7], mouse[12,13], and man [5,8,15,16] As the new sequencing technologies strive toward the capability of sequencing the entire human genome for $1000, these approaches will become routine tools for every facet of gene expression analysis.

In this paper we have focused on the quantification of gene expression levels and have compared the results with DNA microarrays and Quantitative RTPCR using the Microarray Quality Control (MAQC) reference RNA samples[1,2]. We have generated 3.6 million reads of average length 250 bp on Roche's 454 GS FLX for the MAQC A and B reference RNA samples. Using the ExpressSeq pipeline these reads can be easily mapped to the human RefSeq genes on a Windows desktop computer and gene expression levels conservatively determined by simply counting the numbers of unique hits with e-values ≤ 10^-20^.

Using the MACQ quality metrics for evaluating the reproducibility, sensitivity, specificity, and accuracy of the gene expression analysis, we have demonstrated that these ExpressSeq results already compare favourably with the results for DNA microarrays and QRTPCR in the MAQC studies. Moreover, a simple Poisson error model for the random shotgun sequencing process describes how these metrics systematically improve with increased numbers of reads.

The presence of repeated sequences arising from homologous gene families and from LINE, SINE, and ALU elements in untranslated regions of the transcriptome pose a significant challenge to the accurate quantification of gene expression by read counts. This is a problem that is well appreciated by the DNA microarray and QRTPCR communities that have been very careful to try to avoid these problems by carefully designing gene probes and primers. Here we have tried to minimize the problem by counting each read only once. However, quantitative errors from the miss-assignment of this read are inevitable. Better strategies for dealing with reads with multiple alignments are required. For example, reads with multiple equivalent alignments to the genome could be eliminated altogether or reads counted only when they map to gene coding regions or to known exons [13].

We have also used the AceView alignment software[18] to co-align these 3.6 million reads for A and B samples to the human genome to identify over 20,000 new exon junctions in the human transcriptome. Although these candidate exon junctions may be less common than the well-annotated RefSeq junctions, they may still play an important role in determining the diversity of biological phenotypes. These results indicate that there is still a significant amount of biology to be uncovered using transcriptome sequencing.

At present genomics researchers can choose among several next-generation sequencing platforms for transcriptome sequencing that generate either long, 250 bp–400 bp, (Roche GS FLX) or short, ~30 bp, (Illumina Genome Analyzer and ABI SOLiD) reads. The larger numbers of reads generated by the short read platforms provide greater sequencing depth and should provide better reproducibility, sensitivity, specificity, and accuracy for the measurement of differential gene expression. However, the longer reads are easier to map and to assemble. Consequently, the long reads should be better for the discovery and assembly of novel genes and splice variants.

For example, three papers have recently appeared with deep sequencing results for transcriptomes of different human cell lines [21,22] and tissues[22,23] using the Illumina Genome Analyzer. Although these studies generated between 16 million[21] and 435 million[22] reads for expressed transcripts, they succeeded in only identifying between 4096[21] and 11,099[23] new exon junctions not contained in the human Ensembl and RefSeq databases by aligning the short, 32 bp, reads to databases of predicted splice junctions. In particular, the largest study[22] with more than 400 million reads discovered only 114 new "isolated" cassette exons in all of the 15 human tissues and cell lines examined, as compared with the 912 new cassette exons found here by aligning 3.6 million long reads for the MAQC samples directly to the human genome.

A remaining challenge is the proper assembly of the transcribed exons into the full-length alternative transcripts [24]. This will require preservation of the identity of the transcribed strand to distinguish reads from overlapping transcripts (which can be accomplished using a modification of the TSEQ protocol) and longer read lengths and/or paired end reads for the transcriptomes will be necessary to link all of the pieces together. Fortunately, the new GS FLX Titanium upgrade now provides average 400 bp reads and all of the new sequencing platforms have protocols for paired end reads to bridge across the full length transcripts. As read lengths and throughput continue to increase and new sequencing platforms [25-27] and software tools [28] for mapping the reads emerge, the combination of the long and short read technologies may be most effective for exploring the complexity of the transcriptome, where the long reads are used for gene discovery and assembly and the short reads for confirmation and quantification[22].

## Conclusion

Gene expression analysis by transcriptome sequencing using the next-generation sequencing technologies shows great promise. Using the metrics introduced for evaluating the performance of gene expression platforms in the MAQC studies, our ExpressSeq results for gene expression levels of the MAQC reference RNA samples showed excellent specificity, sensitivity, and reproducibility that improve systematically with increasing sequencing depth, and quantitative accuracy that compare favourably with DNA microarrays and Quantitative RT-PCR. In addition, a careful mapping of the long GS FLX reads to the genome using the AceView alignment programs shed new light on the complexity of the human transcriptome including the discovery of thousands of new splice variants.

## Methods

### RNA Samples

Sample A is Universal Human Reference RNA (Stratagene catalog # 740000) from pooled human cell lines and Sample B is FirstChoice Human Brain Reference Total RNA (Ambion catalog # AM6051) from pooled whole brain preparations. The two samples are identical to the total RNA samples used throughout the MAQC studies.

### Sample Preparation

Two different general methods for preparing cDNA for sequencing were compared in this study. [See Additional file 1 for Supplementary Methods for full details]

1) An Oligo DT (ODT) protocol, similar to the process used to prepare samples for DNA microarray studies, was utilized to prepare double stranded cDNA for the standard Roche GS DNA Library preparation and sequencing process. For some sequencing runs modified oligo dT primers ending with two different additional nucleotides were introduced to help eliminate reads with long poly A strings.

2) A Transcriptome Sequencing (TSEQ) protocol using random primers was applied to heat-fragmented mRNA strands to generate a single stranded cDNA library for sequencing using the standard Roche GS Amplicon sequencing process. It was hoped that the use of random primers would remove a potential 3' bias in the ODT preparation.

In all cases, a thorough depletion of rRNA, which can constitute as much as 98% of the total RNA, was required to minimize the numbers of sequencing reads from rRNA contamination, even for the ODT protocol. Several different methods for rRNA depletion were tested for the ODT protocol in different sequencing runs ranging from a simple ribo-reduction step to multiple mRNA enrichment steps while the TSEQ protocol consistently utilized a rigorous 2 step mRNA enrichment using magnetic beads. When multiple rRNA reduction steps were used, the rRNA reads were reduced to less than 10% of the total.

### Sequencing and Signal Processing

The samples were sequenced using the standard GS FLX protocol. The single stranded cDNA libraries (with ligated adapters) are annealed to DNA sequencing beads and then clonally amplified using emulsion PCR (emPCR). Hundreds of thousands of the DNA library beads are deposited onto 44 micrometer diameter wells on a PicoTiterPlate and sequenced by synthesis, where successive incorporation of nucleotides are imaged using chemoluminescence with a CCD camera on the GS FLX instrument. The image analysis, signal processing and data filtering are performed using the standard 454 Software with the "decreased stringency" and "lowest stringency" settings: vfLastFlowToTest = "168" and vfBadFLowThreshold = "6". The lower stringency settings on the read quality filters result in 20–50% more useful reads from the sequencing runs for mapping to the reference sequence databases. Finally, all of the reads that pass the quality filters are deposited into the output files in flowgram (.sff) and FASTA (.fna) formats for further analysis and have been archived at the NCBI Short Read Archive Accession [NCBI:SRA003647].

### ExpressSeq pipeline for read mapping

Eleven full GS FLX sequencing runs were completed for this study for cDNA prepared from the MAQC A and B samples using different protocols described above. Each sequencing run generated up to 500,000 reads on the large LR75 sequencing plates divided into two regions. In most cases the A and B samples were run simultaneously in two regions on the same plate. In a few cases the same sample was run in both regions to compare the inter- and intra-run reproducibility of the sequencing results. [See Additional file 1 for Supplementary Table for a guide to the sequencing runs]

The ExpressSeq pipeline maps each of the long reads to the Human Genome or the RefSeq (Build 36.2), CCDS, and AceView databases of expressed sequences from July 2007 using MegaBlast on Windows desktop computer or TeraBLAST implemented on a TimeLogic DeCypher. The vast majority of the long reads could be mapped unambiguously to the reference databases with default BLAST parameters and e-values ≤ 10^-20 ^or better, corresponding to at least 50 perfect match bases. Several hundred thousand reads could be easily mapped to the 38,698 RefSeq or 17,751 CCDS sequences in a few hours of CPU time on the Windows Desktop while the mapping of all of the 3.6 million reads in this study could be mapped to either the much larger AceView database in an overnight run or to the entire human genome in 6 days using the TimeLogic DeCypher accelerator board.

Expression levels could then be inferred by simply counting the number of reads in each sample that hit each reference sequence. A number of reads were found to hit regions associated with repeat elements such as LINEs, SINEs, and ALUs that may occur even in expressed sequences, especially 3' UTRs. Since these ambiguous hits will skew the inferred gene expression levels, each read was only counted once for the single best hit with smallest e-value. No effort was made here to resolve "ties", only the first "best" hit in the BLAST output files was counted.

Although less than 4% of long reads map ambiguously to multiple locations in the genome with e ≤ 10^-20 ^in all samples and sample preparations, multiple equivalent hits to RefSeq sequences can frequently arise when two or more splice variants of the same gene are present with different accession numbers. Here, the fraction of ambiguous reads increases to 20 – 26%. In these cases only the reads associated with specific exons or exon junctions can be unambiguously assigned to specific splice variants. Consequently, for comparison with the DNA microarray and QRTPCR data, the ExpressSeq pipeline simply sums the hits for all splice variants of the same gene to infer the expression level of the "gene".

The BLAST output files used for the hit counts also provide the start and stop sites of the read alignments to Reference so that the locations of the hits can be determined to assess the 5' to 3' coverage of the GS FLX reads. An Excel spread sheet with the ExpressSeq read counts for the RefSeq NM genes for each sequencing run for Samples A and B is provided [see Additional file 2]

### Read alignment to the genome using AceView

A different approach to read mapping is provided by the AceView software. The AceView database and website provides a curated, comprehensive, and non-redundant sequence representation of all public mRNA sequences (from the GenBank, RefSeq dbEST and Trace databases). This was accomplished by co-aligning all publicly available mRNAs and ESTs onto the genome sequence, using an original cDNA-to-genome coalignment program, initially called Acembly.

Because of the high quality of the long GS FLX reads, with sequencing errors (primarily due to homopolymer insertion and deletions) of less that 1%, these expressed sequences reads could be aligned with exactly the same software settings as used for the public collection of ESTs and cDNAs from Sanger sequencing over the past 15 years.

The best alignment for each read was determined with the stringent requirement that over 90% (on average > 245 bp) of the entire sequence align perfectly to the human genome. When the alignment covered disjoint segments on the same chromosome separated by traditional introns with boundaries defined by GT-AG, GC-AG, or AT-AC (0.05%) with at least 8 consecutive, perfect match bases on each side of the intron, the read was counted as spanning an exon boundary. With these strict requirements, thousands of new exon splice sites were identified including 100's of new cassette exons that had not been previously experimentally observed.

The genomic locations of the 137,899 unique exon junctions and the identities of the supporting reads from the A and B samples are provided in tab delimited format [see Additional file 3]. An Excel Spreadsheet containing the locations and supporting reads for the candidates for new cassette exons and skipped exons is provided [see Additional file 4]. This file also contains a list of diseases associated with the genes where these new splicing events are found.

### Affymetrix exon arrays

The MAQC A and B RNA reference samples were analyzed using the Affymetrix Human Exon 1.0 GeneChips using standard Affymetrix protocols and the data analyzed using the RMA algorithm in the Affymetrix Expression Console software to identify differential expression at the exon level for comparison with the ExpressSeq results[29].

### Accession numbers

All transcriptome sequencing data from this study are available through the NCBI Short Read Archive Accession number [NCBI:SRA003647] including the raw standard flowgram files (SFF) and the read fasta (FNA) files. The Affymetrix Exon array data is available at GEO Accession number [NCBI:GSE13072].

## Competing interests

TH and SKH are both employees of Roche. There are on other competing interests.

## Authors' contributions

CE, SH, and KLC performed sample preparation and sequencing. SPM, DTM, JTM and RVJ carried out the Data analysis. OC, OF, and TH helped to coordinate the execution of the project and edit the manuscript. SPM, DTM, JTM, and RVJ wrote the manuscript. All authors have read and approved the final manuscript.

## Supplementary Material

Additional file 1 **Supplementary Materials**. Contains detailed **Supplementary Methods **for the preparation of the samples for Transcriptome Sequencing, the **Supplementary Analysis **for determining the scaling properties of the depth of coverage curves, the **Supplementary Figure **comparing the relative accuracy of the ExpressSeq results with different microarray platforms, and the **Supplementary Table **containing the guide to the different sequencing runs and data files. Click here for file

Additional file 2 **ExpressSeq Read Counts for the A and B Samples**. An Excel Workbook provides the NM "hit" counts for each of the 22 sequencing regions on the 11 full GS FLX sequencing plates described in **Supplementary Table 1 **in Additional file 1. The Excel file contains 4 Worksheets providing the hit counts for the sequencing runs for the A and B samples processed using either the TSEQ or ODT protocols. The first 2 columns of each Worksheet contain the RefSeq NM number and description. The third column provides the RefSeq transcript length and the subsequent columns give the numbers of reads that hit each transcript with evalues < 1.0e^-20 ^for each sequencing region. Click here for file

Additional file 3 **All Standard Exon Junctions**. This compressed tab delimited text file contains the genomic locations (by chromosome number and position) of each of the 137,899 standard exon junctions identified by the stringent AceView alignment of the 3.6 million reads for the MAQC A and B samples generated in this study. In addition, the supporting reads are listed for each junction using the unique alphanumeric names for each sequencing run. [See the **Supplementary Table of **Additional file 1 for a guide.] Click here for file

Additional file 4 **New Cassette Exons and Skipped Exons**. The first Worksheet of this MS Excel file contains the genomic locations (by chromosome number and position) and the identities of the supporting reads for each end of the 912 new cassette exons found inside of a RefSeq intron identified by the stringent AceView alignment of the 3.6 million reads for the MAQC A and B samples generated in this study. The second Worksheet contains the locations of the 249 novel candidate cassette exons that had not been previously identified by either RefSeq or AceView. The third and fourth Worksheets provide the genomic locations and supporting reads of the 504 new skipped exons absent in RefSeq and the 192 novel skipped exons missing from both RefSeq and AceView. The sequences for the supporting reads for each exon boundary can be found in the Short Read Archives Accession Number [NCBI:SRA003647] with the assistance of the **Supplementary Table **in the Additional file 1. The last two Worksheets provide a list of diseases associated with the gene loci where these new cassette and skipped exons are found. Click here for file
