# Supplementary material for: Transcriptome sequencing of the Microarray Quality Control (MAQC) RNA reference samples using next generation sequencing
Source: BMC Genomics. 2009 Jun 12;10:264. doi: 10.1186/1471-2164-10-264 (PMC2707382; doi:10.1186/1471-2164-10-264)
Supplement: Additional file 1 — Supplementary Materials. Contains detailed Supplementary Methods for the preparation of the samples for Transcriptome Sequencing, the Supplementary Analysis for determining the scaling properties of the depth of coverage curves, the Supplementary Figure comparing the relative accuracy of the ExpressSeq results with different microarray platforms, and the Supplementary Table containing the guide to the different sequencing runs and data files. [file 1471-2164-10-264-S1.doc]

**Supplementary Methods**

***Final ODT Preparation for 454 Sequencing.***

20 μg of the two MAQC reference samples were treated with RiboMinus Human/Mouse module (Invitrogen; cat 45-7012 ) according to the makers instruction. This depleted the ribosomal component to 4%. The RNA was then taken through two rounds of mRNA enrichment using Dynal Dynabeads (Cat 610.06) according to the manufacturer’s instruction. Two μl of 50uM Oligo-(dT)_18_-dN_2_ primers (where N_2_ is all combinations of the nucleodite dimers) were annealed to the RNA (65C 5mins 25C 10 min.). The primer annealed RNA was then taken through First Strand synthesis . First Strand synthesis buffer contained 10 mM DTT, 500 μM dNTPs and 8 units of SuperScript II (Invitrogen Life Technologies). This was followed by Second Strand synthesis using the following reagents:

RNase-free Water 91 μL

5X 2nd Strand Reaction Mix 30 μL

dNTP, 10 mM 3 μL

E. coli DNA ligase 1 μL

E. coli DNA Polymerase I 4 μL

RNase H 1 μL

Total Volume 130 μL

The samples were mixed well by gently flicking the tube a few times and then centrifuging briefly for 5 seconds to collect the solution at the bottom of the tube. 130 μL of Second-Strand Master Mix was added to each first-strand synthesis samples for a total volume of 150 μL. The samples were incubated for 2 hours at 16°C. To each sample 4 units of T4 DNA polymerase was added and incubated at 16°C for 5 minutes. After incubation with T4 DNA Polymerase, 10 μL of 0.5M EDTA was added. The samples were cleaned with Qiagen PCR clean up kit to remove double stranded cDNA.

The quality of cDNA was assessed on the Agilent Bioanalyzer and quantified using the Nano Drop spectrometer. Four μg of cDNA was taken through the 454 Roche Library prep with nebulisation. Emulsion PCR was carried out in accordance with 454 Roche’s protocols. The subsequent enriched beads were deposited on the PicoTiterPlate and the cDNA was sequenced using 454 Roche’s GS FLX sequencer.

■ SuperScript™ II, Invitrogen Life Technologies, P/N 18064-014 or SuperScript Choice

System for cDNA Synthesis, Invitrogen Life Technologies, P/N 18090-019

■ *E. coli* DNA Ligase, Invitrogen Life Technologies, P/N 18052-019

■ *E. coli* DNA Polymerase I, Invitrogen Life Technologies, P/N 18010-025

■ *E. coli* RNaseH, Invitrogen Life Technologies, P/N 18021-071

■ T4 DNA Polymerase, Invitrogen Life Technologies, P/N 18005-025

■ 5X Second-strand buffer, Invitrogen Life Technologies, P/N 10812-014

■ 10 mM dNTP, Invitrogen Life Technologies, P/N 18427-013

■ 0.5M EDTA

***TSEQ Library Preparation for 454 Sequencing***

Poly-A+ RNA was prepared from 50 µg total RNA by two rounds of purification with oligo(dT) magnetic beads (PureBiotech, Middlesex, NJ) to minimize ribosomal RNA contamination.  Poly-A+ RNA quality was assessed by Agilent Bioanalyzer RNA 6000 Nano assay (Agilent Technologies, Inc., Santa Clara, CA) and quantified by Quant-iT RiboGreen assay (Invitrogen, Carlsbad, CA).

Poly-A+ RNA (100 ng per sample) was subjected to heat fragmentation to generate fragments compatible with sequencing on the Genome Sequencer FLX (Roche/454 Life Sciences, Branford, CT). Briefly, 20 μl of the RNA sample (in 10 mM Tris-HCl pH 7.5) was mixed with 5 μl 5× Fragmentation Buffer (200 mM Tris-Acetate, 500 mM potassium acetate and 157.5 mM magnesium acetate with final pH adjusted to 8.1) and incubated at 82^o^C for 2 min. The reaction was stopped by immediately transferring the samples to ice post incubation. To recover the fragmented RNA, 10 mM Tris-HCl (pH 7.5) was added to a total volume of 50 μl. After addition of 80 μl (equal to 1.6× the volume of sample) RNAClean solution (Agencourt, Beverly, MA), the mixture was incubated at room temperature for 10 min and the beads were washed as per the manufacturer’s instructions. The fragmented RNA samples were eluted in 10 μl 10 mM Tris-HCL (pH 7.5).

For sscDNA generation, each sample was mixed with 2 μl 500 μM random primer (5’-phosphate-N_7_-OH-3’; Integrated DNA Technologies, Coralville, IA), incubated at 70^o^C for 10 minutes and immediately transferred to ice for 2 minutes. Subsequently, the RNA was reverse transcribed in a total volume of 20 μl of 1× First Strand Synthesis buffer containing 500 μM dNTPs, 10 mM DTT, 10 units of SUPERase-In RNase inhibitor (Ambion, Austin, TX) and 200 units of Superscript II enzyme (Invitrogen Corporation, Carlsbad, CA). The samples were incubated at 25^o^C for 5 minutes followed by 37^o^C for 60 minutes. After addition of 20 μl denaturing solution (0.5 M NaOH, 0.25 M EDTA), the samples were incubated at 65^o^C for 20 minutes to hydrolyze the RNA. The mixtures were neutralized by adding 0.5 M HCl in 0.% M Tris-HCl (pH 8.0) to a pH between 7.0 and 8.5. The resultant sscDNA was recovered with RNAClean solution (1.6× sample volume) following the manufacturer’s instructions and eluted in 12 μl of 10 mM Tris-HCl (pH 7.5).

Adaptors containing 454 sequencing-specific sequences were ligated to the 5’ and 3’ ends of the sscDNA as described below. The adapters are each comprised of two complimentary strands with random hexamer overhangs. The 3’ adapter is generated by annealing oligo A-prime 5’-Bio-GCCTTGCCAGCCCGCTCAGNNNNNN -Phos- 3’ to oligo A 5’-Phos- CTGAGCGGGCTGGCAAGG –dideoxyC- 3’ and the 5' adapter is generated by annealing oligo B 5’ – NNNNNNCTGATGGCGCGAGGGAGG –dideoxyC- 3’ to oligo B-prime 5’ – GCCTCCCTCGCGCCATACG – 3’ using the following annealing protocols: 50 µM oligo A and 60 µM oligo A-prime (3’ adapter) or 240 µM oligo B and 200 µM oligo B-prime (5’ adapter) was combined in 100 µl Tris-HCl (pH 7.5) and incubated in a thermocycler for 5 min at 80^o^C, 7 min at 65^o^C, 7 min at 60^o^C, 7 min at 55^o^C, 7 min at 50^o^C, 7 min at 45^o^C, 7 min at 40^o^C, 7 min at 35^o^C, 7 min at 30^o^C, 7 min at 25^o^C, hold at 4^o^C.

The ligation reactions were carried out in a total volume of 30 μl containing 12 µl of the eluted sscDNA, 1X Quick Ligase buffer (New England Biolabs, Ipswich, MA), 1.67 µM of the 5' adapter, 6.67 µM of the 3' adapter and 2000 units of T4 DNA Ligase (New England Biolabs, Ipswich, MA) at 37°C for 2 hours. The reactions were terminated by addition of 70 µl of 1X TE (pH 8.0). Ligated material was recover by adding 100 µl of binding and wash buffer (10 mM Tris-HCl pH 7.5, 1 mM EDTA pH 8.5, 2 M NaCl) containing 0.05 % Sera-Mag 30 beads (Seradyn Inc, Indianapolis, IN) and mixing for 15 minutes at room temperature. Unbound material was washed away and the ligated products were eluted by two successive 45 µl elutions with bead elution buffer (25 mM NaOH, 1 mM EDTA pH 8.0, 0.1 % Tween‑20), pooling both recovered amounts. The final sscDNA libraries were subjected to two rounds of purification with 1.6× sample volume RNAClean bead mix following the manufacturer’s directions . The purified adapted sscDNA libraries were eluted in 12 µl of 10 mM Tris-HCl (pH 7.5).

Size distributions of the final sscDNA libraries were confirmed by RNA 6000 Pico Chip assay (Agilent Technologies) and quantification was performed with the Quant‑iT Ribogreen RNA Assay Kit. Subsequently, each library was diluted to a working concentration for clonal emulsion PCR.

**Supplementary Analysis**

***Scaling of Coverage Curves***

The coverage curve,$C_{n}(N)$, for the number of transcripts detected with at least *n* hits among *N* reads is determined by the integral,

$$C_{n}(N)=\int_{0}^{\infty} Q(\lambda)f(\lambda,n,N)d\lambda,$$

where $Q(\lambda)$ is the distribution of Poisson detection frequencies for transcripts in the sample and $f(\lambda,n,N)=1-\sum_{k=0}^{n-1} e^{-N\lambda}(N\lambda)^{k}/k!$ is the cumulative density of the

Poisson distributions for at least *n* hits for a transcript with detection frequency $\lambda.$

For each transcript the detection frequency by shotgun sequencing will be dependent on the relative abundance of the gene, the length of the gene, and the uniformity of the sequencing coverage. Although the distribution $Q(\lambda)$is not known *a priori* it is expected that there will be a large number of transcripts with low abundance and small values of $\lambda$ and relatively few transcripts of high abundance and large values of $\lambda$.

Since the cumulative density approaches $f(\lambda,n,N)\to\left\{\begin{matrix} 0,N\lambda<n, \\ 1,N\lambda>n. \end{matrix}\right.$ for large values of *n* and *N* with *n/N* fixed, the integral for the coverage curve can be approximated by,

$$C_{n}(N)\approx\int_{n/N}^{\infty} Q(\lambda)d\lambda,$$

which explicitly displays the scaling property, *C_n_(N) ~ C_n/x_(N/x)*, used to generate predicted coverage curves in **Fig. 1**. Note that this approximate formula also indicates how the distribution of detection frequencies can be estimated from the empirical data by simply evaluating the derivative of $C_{n}(N)$ with respect to *n/N.*

**Supplementary Figure**


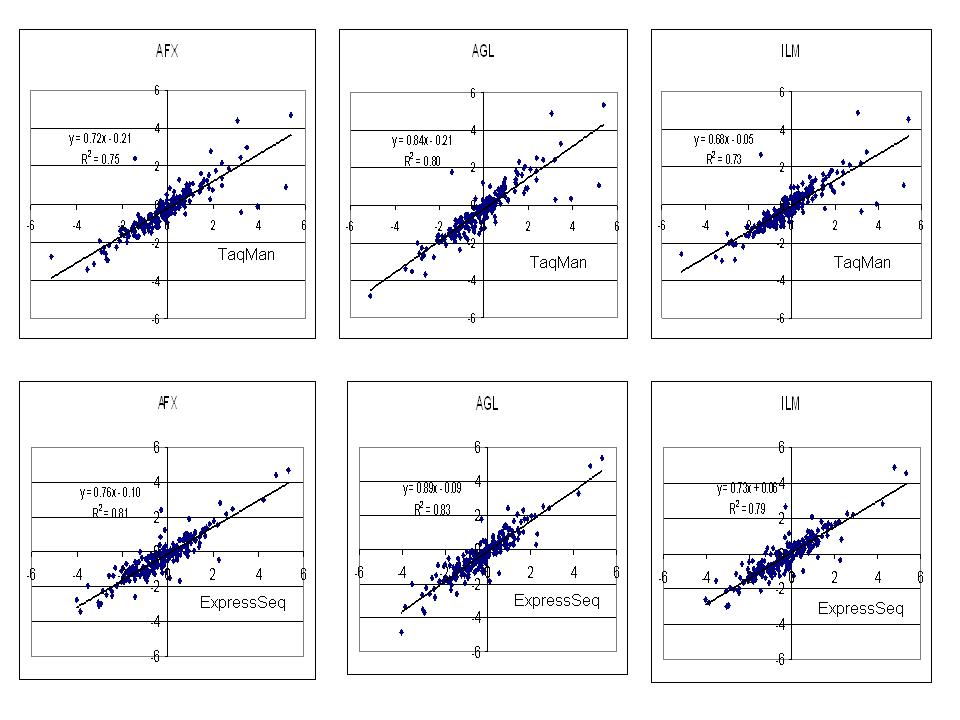


***Comparison with the Accuracy of the MAQC microarray results.*** In the main text **Fig. 3b** shows a comparison of the ExpressSeq and QRTPCR measurements of the fold-changes (on log base 2 scales) between the A and B sample for the 217 genes found to be “present” in the MAQC TaqMan assays and on all of the major microarray platforms (ABI, Affymetrix, Agilent, GE, and Illumina). The Pearson correlation for the ExpressSeq results of R^2^ = 0.81 compares very well with the correlation for the MAQC microarray results with TaqMan for the same 217 genes shown in the top 3 panels of the **Supplementary Figure**, Affymetrix (0.75), Agilent (0.80), and Illumina (0.73). In addition the slope of the trend line in **Fig. 3b** is 0.87 indicating very little compression of fold changes compared with QRTPCR. In comparison the trend line slopes for the DNA microarrays shown in the top three panels of the **Supplementary Figure** are ABI Affymetrix (0.72), Agilent (0.84), and Illumina (0.68). The lower three panels in the **Supplementary Figure** shows a direct comparison of the fold-changes from the MAQC microarray results with the ExpressSeq measurements for the same 217 “present” genes in the A and B samples. It is interesting to note that in every case the Pearson correlation between the microarrays with the transcriptome sequencing is even better (0.76), Affymetrix (0.81), Agilent (0.83), and Illumina (0.79) than with QRTPCR, suggesting that the transcriptome sequencing of the A and B samples may establish a new “gold standard” for gene expression measurements.

# Supplementary Table

| **Sample Code** | **MAQC Sample** | **Method** | **GS FLX Region Code** | **Number of Reads** |
| --- | --- | --- | --- | --- |
| **JAA1** | **A** | **TSEQ** | **ER85B5301** | **122,000** |
| **JAA2** | **A** | **TSEQ** | **ESJ6S1K01** | **141,000** |
| **JAA3** | **A** | **TSEQ** | **E4QP0ZC02** | **128,000** |
| **JAA4** | **A** | **TSEQ** | **E6X9J9101** | **226,000** |
| **JAA5** | **A** | **TSEQ** | **E6X9J9102** | **207,000** |
| **JAB1** | **B** | **TSEQ** | **ER85B5302** | **109,000** |
| **JAB2** | **B** | **TSEQ** | **ESJ6S1K02** | **131,000** |
| **JAB3** | **B** | **TSEQ** | **E4QP0ZC01** | **91,000** |
| **JAB4** | **B** | **TSEQ** | **E6138SN01** | **249,000** |
| **JAB5** | **B** | **TSEQ** | **E6138SN02** | **265,000** |
| **JAB6** | **B** | **TSEQ** | **E56FR8H01** | **120,000** |
| **JAB7** | **B** | **TSEQ** | **E56FR8H02** | **115,000** |
| **JOA1** | **A** | **ODT** | **ERDI6OO02** | **138,000** |
| **JOA2** | **A** | **ODT** | **ERTYMEZ02** | **227,000** |
| **JOA3** | **A** | **ODT** | **E3VCB0K02** | **209,000** |
| **JOA4** | **A** | **ODT** | **E5W9ZWM01** | **192,000** |
| **JOA5** | **A** | **ODT** | **E5W9ZWM02** | **116,000** |
| **JOB1** | **B** | **ODT** | **ERDI6OO01** | **89,000** |
| **JOB2** | **B** | **ODT** | **ERTYMEZ01** | **292,000** |
| **JOB3** | **B** | **ODT** | **E3VCB0K01** | **199,000** |
| **JOB4** | **B** | **ODT** | **E54PARH01** | **124,000** |
| **JOB5** | **B** | **ODT** | **E54PARH02** | **142,000** |

# *Guide to Sequencing Results for the MAQC A and B samples.* Eleven full GS FLX sequencing runs were performed for the MAQC A and B samples on the large LR75 sequencing plate divided into 2 sequencing regions. Supplementary Table 1 provides a guide to the sequencing run and read nomenclature for used in Additional Files 2, 3 and 4 and for the NM “hit”counts and tables of splice junctions and for the raw data .sff and .fna files deposited in NCBI Short Read Archive Accession [NCBI:SRA003647.1]. The first column contains a simple four character name for each sequencing region used by the authors to distinguish the different data files, the last letter in the code refers to the sample type, MAQC A and B, and the next to last letter refers to the sample preparation method, O for ODT and A for TSEQ, listed in the second and third columns. The fourth column contains the unique 9 character alphanumeric GS FLX code for the sequencing region that is used to name the corresponding .sff and .fna files and provides the prefix for unique read names from that sequencing region. The fifth column provides the total number of reads that passed the GS FLX quality filters for that region.
